# High agreement of three‐dimensional lower limb alignment analysis between a novel cone‐beam CT and multislice CT

**DOI:** 10.1002/jeo2.70539

**Published:** 2025-11-14

**Authors:** Philipp Blum, Julius Watrinet, Marianne Hollensteiner, Peter Augat, Michael Scherr, Michael Seidenbusch, Marcus Treitl, Fabian Stuby, Julian Fürmetz

**Affiliations:** ^1^ Department of Trauma Surgery BG Unfallklinik Murnau Murnau Germany; ^2^ Institute for Biomechanics BG Unfallklinik Murnau Murnau Germany; ^3^ Department of Orthopaedic Sports Medicine Technical University of Munich Munich Germany; ^4^ Institute for Biomechanics Paracelsus University Salzburg Salzburg Austria; ^5^ Department of Radiology BG Unfallklinik Murnau Murnau Germany; ^6^ Department of Orthopaedics and Trauma Surgery University Hospital, LMU Munich, Musculoskeletal University Center Munich (MUM) Munich Germany

**Keywords:** 3D imaging, alignment, cone beam computed tomography, lower limb, Multitom Rax

## Abstract

**Purpose:**

Lower limb alignment analysis based on three‐dimensional (3D) models has become integral to preoperative planning. However, 3D models are typically reconstructed from data acquired in non‐weight‐bearing conditions. This study aims to validate a cone beam computed tomography (CBCT) for a three‐dimensional assessment of the lower limb in upright position.

**Methods:**

A fixed phantom limb, derived from supine CT data, was built and scanned five times by two different imaging modalities (CBCT and multislice computed tomography (MSCT)) in the upright and supine positions. A surface comparison between the 3D models generated from CBCT and MSCT was conducted. Furthermore, two observers performed alignment analysis by determining 31 landmarks on each 3D model. Intra‐ and interobserver variability of the landmarks and root mean square error (RMSE) of angles, axes, patellofemoral values, and leg lengths were evaluated.

**Results:**

The surface comparison revealed excellent agreement, with deviations primarily confined within the range of ±0.2 mm. Intra‐ and interobserver variability of all landmarks was less than 5 mm in both imaging modalities. The mean precision of all landmarks in CBCT and MSCT showed no statistically significant difference between both modalities (1.38 mm [SD 0.63] vs. 1.46 mm [SD 0.74], *p* = 0.444). For angles and axes, interobserver mean values in MSCT and CBCT differed by less than one degree in all measurements except femoral torsion (>2°). RMSE for angles and axes was less than one degree in most measurements except for tibial and femoral torsion.

**Conclusion:**

CBCT and MSCT showed a high level of agreement in 3D imaging of the lower limb and assessing alignment and joint level metrics. By offering 3D imaging under physiological load, CBCT may have the potential to improve preoperative planning in clinical use.

**Level of Evidence:**

N/A.

## Graphical abstract

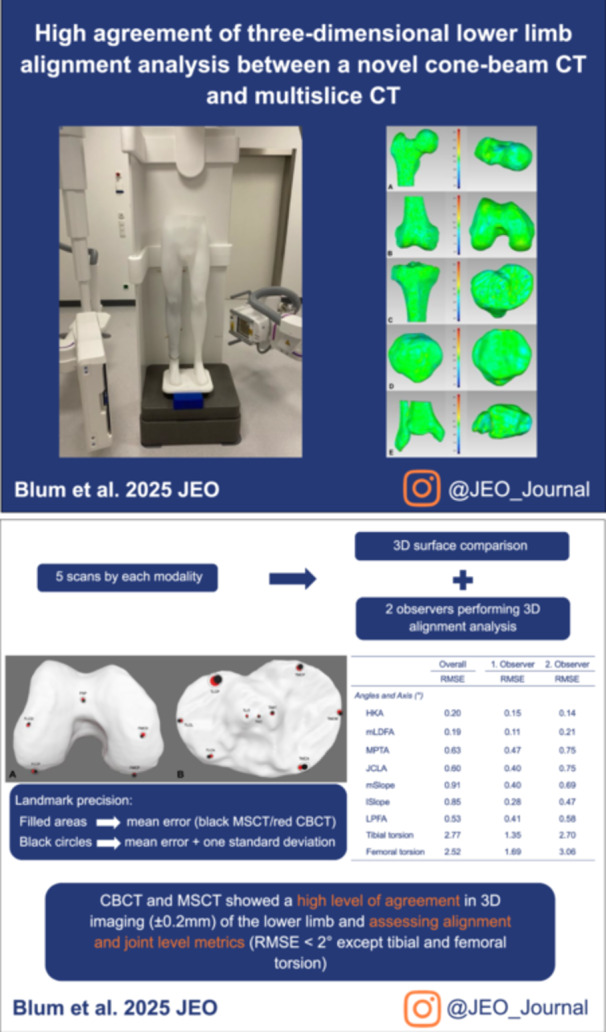

**Abbreviations**
CBCT: cone bean computed tomography
FLCD: femoral lateral condyle distal
FLCP: femoral lateral condyle posterior
FMCD: femoral medial condyle distal
FMCP: femoral medial condyle posterior
FNP: femoral notch point
FT: femoral torsion
HKA: hip‐knee‐ankle angle
ISI: Insall–Salvati index
lSlope: lateral posterior slope
JLCA: joint line convergence angle
LL: leg length
LPFA: lateral proximal femoral anlge
mLDFA: mechanical lateral distal femoral angle
MPTA: medial proximal tibial angle
MSCT: multislice computed tomography
mSlope: medial posterior slope
RMSE: root mean square error
SD: standard deviation
TLCA: tibial lateral condyle anterior
TLCL: tibial lateral condyle lateral
TLCP: tibial lateral condyle posterior
TLIT: tibial lateral intercondylar tubercle
TMCA: tibial medial condyle anterior
TMCL: tibial medial condyle lateral
TMCP: tibial medial condyle posterior
TMIT: tibial medial intercondylar tubercle
TT: tibial torsion
TTTG: tibial tuberosity‐trochlear groove distance

## INTRODUCTION

The alignment of lower limb bones pertains to the precise anatomical positioning of the femur, tibia, and fibula, which is critical for maintaining biomechanical efficiency and optimal load distribution during movement. For deformity correction or joint replacement procedures, an accurate preoperative assessment of the leg axis on weight‐bearing radiographs is essential for a successful surgical procedure and long‐term functional success [19]. However, factors such as load [29], knee flexion [16] and rotation [6, 21] have been shown to influence measurement accuracy. In particular, studies indicated that increased internal rotation is associated with a linear decrease of the measured hip‐knee‐ankle angle (HKA), with its effect being further amplified by additional knee flexion [1, 21]. As a result, this method is prone to potential errors [25].

In recent years, the use of three‐dimensional (3D) bone models in combination with patient‐specific instruments (PSI) and robotics in orthopaedic surgery have gained popularity due to a more precise understanding of the patient's individual anatomical features [7, 23, 24, 30]. These 3D models are predominantly derived from multislice computed tomography (MSCT) scans performed in a supine, non‐weight bearing position. Previous studies have demonstrated that essential planning parameters, such as the HKA and the joint line convergence angle (JLCA), differ significantly between 2D weight‐bearing images and 3D non‐weight bearing images [14, 22], resulting in an underestimation of deformities in non‐weight bearing models [22].

Dedicated imaging systems such as EOS‐Imaging (EOS imaging, Paris, France) allow for weight‐bearing, whole‐leg axis imaging using synchronized perpendicular X‐ray beams, enabling algorithm‐based 3D reconstructions of the lower limb for measuring parameters such as tibial and femoral torsion [9]. Despite its advantages in diagnosing deformities, the software (ster EOS, EOS imaging, Paris, France) generates 3D models by interpolating missing data through optimization of a shape model [3, 4]. Therefore, the reconstructed 3D models do not precisely match the actual bony surface, making them unsuitable for 3D surgical planning and the development of PSI.

Another modality gaining popularity in musculoskeletal imaging in recent years is cone‐beam computed tomography (CBCT), exemplified by systems such as the Multitom Rax (Siemens Healthineers, Forchheim, Germany). This x‐ray room solution is equipped with two‐ceiling mounted robotic arms that can independently move and rotate the X‐ray source and the flat‐panel detector, allowing for flexible imaging configurations, including 2D radiography and 3D CBCT under weight‐bearing conditions. However, until now, it has not been possible to image multiple joints with this system while preserving spatial orientation relative to each other. Consequently, there is a lack of information on whether this imaging technique is suitable for three‐dimensional long leg alignment analysis.

Therefore, the aim of this study was to compare the level of agreement in leg axis analysis between 3D reconstructions obtained using the CBCT and the MSCT technique. It was hypothesized that identical 3D models could be derived from each imaging modality, allowing for equivalent alignment analysis.

## METHODS

### Limb phantom

**Figure 1:**
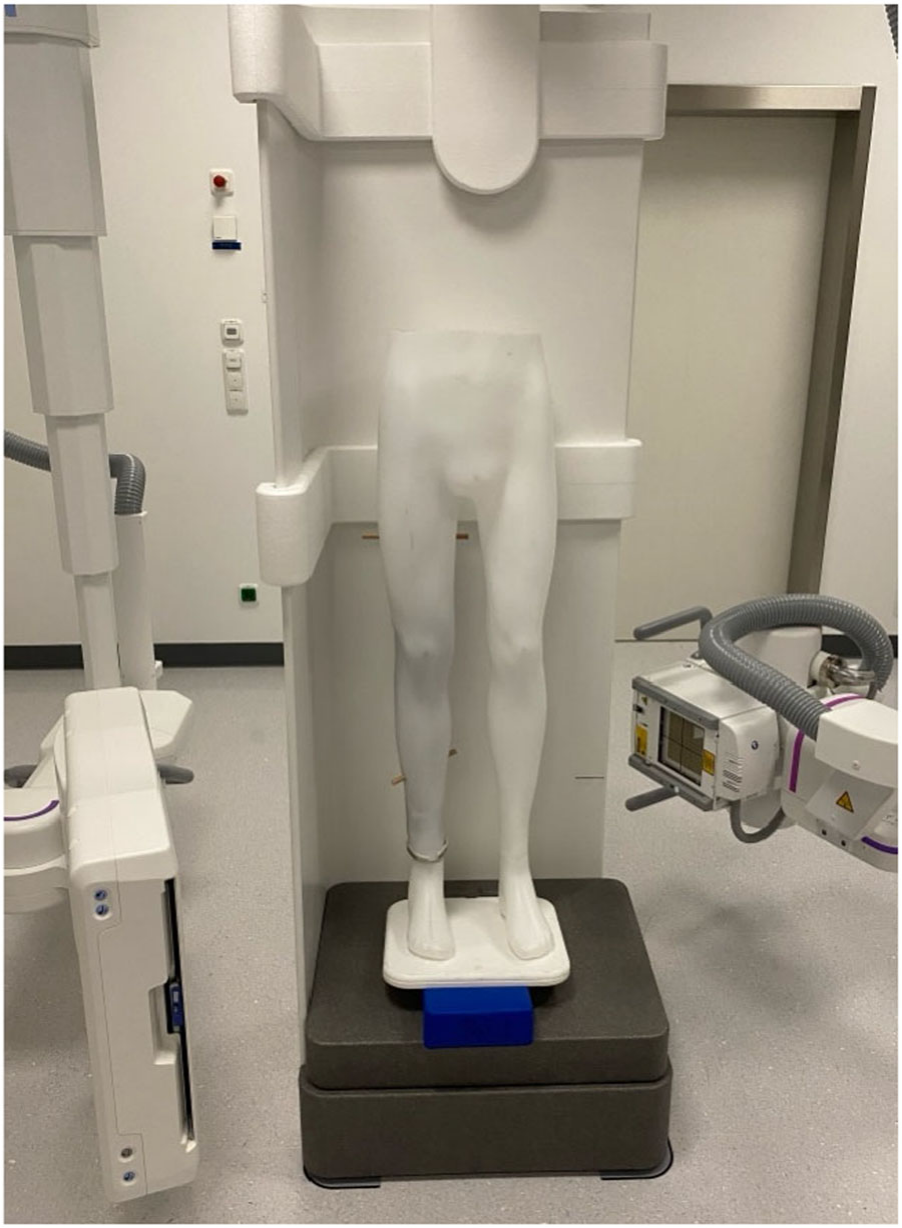
Illustration of the limb phantom standing in an upright position in the cone bean computed tomography (CBCT) device.

For this study, fully anonymized pre‐existing CT data of the entire lower limb of a man was used. To obtain a 3D model of the femur, tibia, fibula, and patella, the data set was segmented using 3D Slicer (https://www.slicer.org/, version 5.6.2) [8]. Afterwards, the surface was smoothed, and holes were filled using the same software. The processed data were prepared for 3D printing via Simplify 3D (version 5.1.2, Cincinnati, Ohio, USA), and a 3D printer (N2‐plus, Raise 3D, Irvine, USA) was then utilized to print a model of the specified bones. The printed parts of the lower limb were assembled in an anatomical alignment using carbon fiber, which was selected for its high rigidity and radiolucency to ensure structural stability without introducing imaging artifacts. Foam was employed around the knee joint to maintain a physiological joint gap between the femur and tibia. Additionally, the patella was also embedded in the foam to mimic its anatomical location. Both materials were carefully chosen to replicate anatomical alignment while minimizing interference with image quality in CBCT and MSCT scans, without compromising the surface integrity of the periarticular components required for accurate 3D reconstruction. Finally, the entire model was placed inside a mannequin and fixed with wooden pins inserted in the femur and tibial shaft to achieve an upright position. The limb phantom is shown in Figure 1.

### Radiological scans

**Figure 2:**
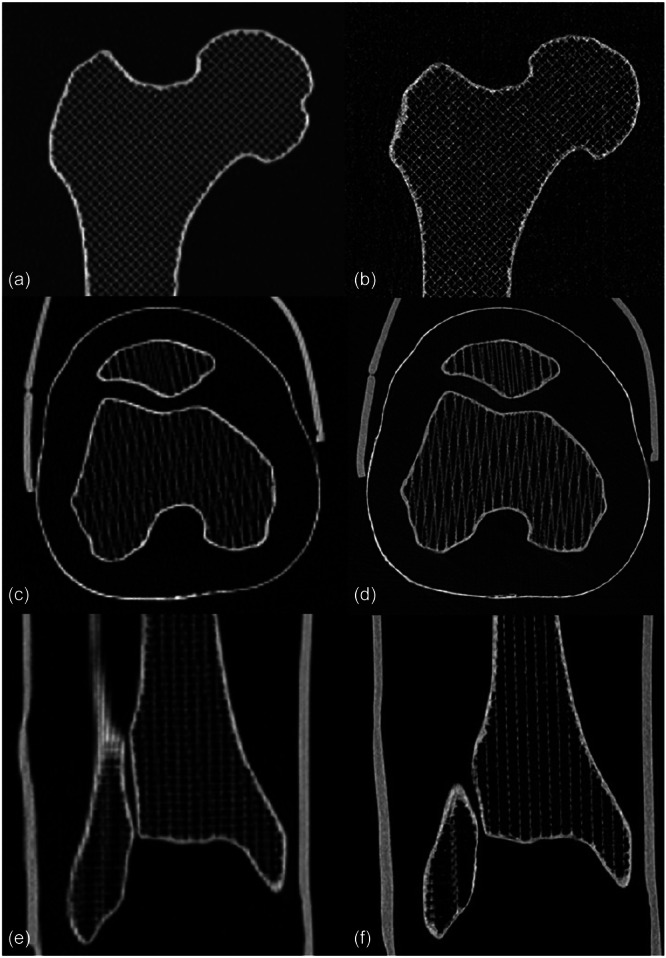
Images of the hip (coronal view), knee (axial view) and ankle joints (coronal view) of the limb phantom using multislice computed tomography (MSCT) (a, c, e) and cone bean computed tomography (CBCT) (b, d, f).

Radiological scans were first performed in 3D CBCT mode on the Multitom Rax (Siemens Healthineers, Forchheim, Germany). The limb phantom was positioned in an upright position with the patella pointing anteriorly. Short scans of the hip, knee and ankle joints were performed in a standardized procedure using manufacturer settings (hip: kV 72.9, W/L 2000/300; knee: kV 115.4, W/L 2000/300; ankle: kV 115.4, W/L 2000/300) resulting in a reconstructed slice thickness of 0.35 mm in sharp kernel. The clinical scan protocol captured the absolute position, thereby enabling the calculation of the spatial relationships between the various anatomical components. In a second step, the limb phantom was scanned in supine position with the MSCT technique on the SOMATOM Definition AS (Siemens Healthineers, Forchheim, Germany) by using a standard protocol for torsional measurements of the lower limb (hip: kV 140, W/L 2500/550; knee: kV 120, W/L 2500/550; ankle: kV 120, W/L 2500/550) with a slice thickness of 0.6 mm, routinely used in clinical practice. The limb phantom was removed and repositioned repeatedly after each scan by an orthopaedic surgeon until a total of 5 scans were conducted by each modality. All radiological acquisitions were performed by two radiologic technologists who had been trained in the operation of both imaging systems. MSCT and CBCT images of the limb phantom are shown in Figure 2.

### 3D anatomy analysis

**Figure 3:**
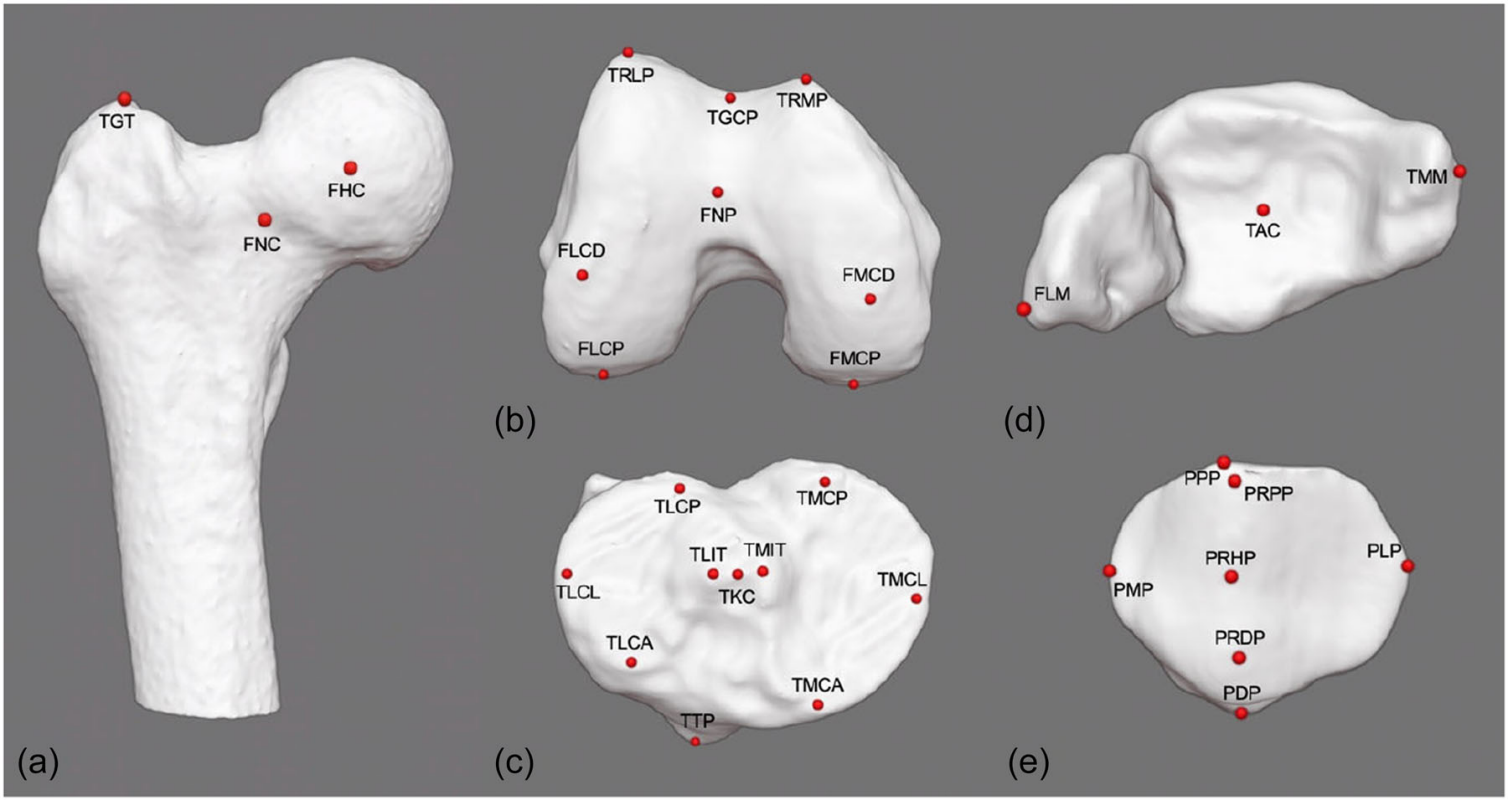
3D reconstruction by CBCT of (a) proximal femur, (b) distal femur, (c) proximal tibia, (d) ankle and (e) patella showing all 31 bony landmarks. CBCT, cone bean computed tomography; FHC, femoral head center; FLCD, femoral lateral condyle distal; FLCP, femoral lateral condyle posterior; FLM, fibular lateral malleolus; FMCD, femoral medial condyle distal; FMCP, femoral medial condyle posterior; FNC, femoral neck center; FNP, femoral notch point; PDP, patella distal pole; PLP, patella lateral pole; PMP, patella medial pole; PPP, patella proximal pole; PRDP, patella ridge distal pole; PRHP, patella ridge high point; PRPP, patella ridge proximal pole; TAC, tibial ankle center; TGT, tip of greater trochanter; TKC, tibial knee center; TLCA, tibial lateral condyle anterior; TLCL, tibial lateral condyle lateral; TLCP, tibial lateral condyle posterior; TLIT, tibial lateral intercondylar tubercle; TMIT, tibial medial intercondylar tubercle; TMCA, tibial medial condyle anterior; TMCM, tibial medial condyle medial; TMCP, tibial medial condyle posterior; TMM, tibial medial malleolus; TGCP, trochlear groove central point; TRLP, trochlear ridge lateral point; TRMP, trochlear ridge medial point; TTP, tibial tuberosity point.

Data from each CBCT and MSCT scan were manually reconstructed using 3D Slicer (https://www.slicer.org/, version 5.6.2). The finalized 3D models were subsequently imported into Blender (Blender Foundation, Amsterdam, Netherlands, version 4.1). Both software tools are free, open‐source programs. An experienced orthopaedic surgeon established the coordinate system for each model in accordance with former publications. The x‐axis was defined by fitting a cylinder through both femoral condyles and the z‐axis was determined as a line perpendicular passing through the center of the femoral head [15], allowing for a comparison of the coordinates across models. Subsequently, two experienced orthopaedic surgeons identified 31 bony landmarks on each 3D model resulting in a total number of 20 sets of coordinates (Figure 3). These landmarks were previously defined by Fürmetz et al. and had shown low inter‐ and intraobserver variability in prior studies [10, 11]. A custom‐made Python script in Blender facilitated the automatic calculation of angles and axes based on the coordinates of the bony landmarks. Thereby, angles and axes were defined as previously described [10, 11]. All orthopaedic surgeons had extensive experience in 3D lower limb analysis and had previously performed measurements based on predefined anatomical landmarks outside the scope of this study.

### Outcome measurements

A surface comparison between the 3D models generated from CBCT and MSCT scans was conducted using Geomagic Wrap 2021 (3D Systems, Rock Hill, USA). Therefore, two 3D reconstructions from each imaging modality were aligned to one another by using the best‐fit surface alignment tool of the software, which superimposes the surfaces based on geometry alone. Geomagic then calculated the maximum and mean distances (positive and negative differences) between the two models. These values were visually displayed with a color map by showing the distances between the models in different colors, enabling an objective and landmark‐independent evaluation of surface discrepancies between 3D models of both imaging modalities.

Furthermore, x‐, y‐ and z‐coordinates of each bony landmark were identified. For intraobserver precision, mean landmark position $\overset{®}{P}$ (x, y, z) as well as distances (*D_i_*) to this mean position were calculated according to the following formula of Victor et al. [27]:

$$x=\frac{x_{1}+x_{2}+x_{3}+x_{4}+x_{5}}{5}$$

$$\overset{®}{P}=\frac{P_{1}+P_{2}+P_{3}+P_{4}+P_{5}}{5}\to y=\frac{y_{1}+y_{2}+y_{3}+y_{4}+y_{5}}{5}\;D_{i}=∥\bar{P}-P_{i}∥=\sqrt{{\left(\overset{®}{x}-x_{i}\right)}^{2}+{\left(\overset{®}{y}-y_{i}\right)}^{2}+{\left(\overset{®}{z}-z_{i}\right)}^{2}}$$

$$z=\frac{z_{1}+z_{2}+z_{3}+z_{4}+z_{5}}{5}$$

To quantify intraobserver variability for a specific landmark, mean value and standard deviation (SD) of *D_i_* were calculated.

The interobserver precision was represented as the mean position of landmarks by using the means of coordinates found by the two observers. Interobserver variability was assessed by the mean and SD of *D_i_* relative to the aforementioned mean positions [27].

The following angles and axes were calculated: [11] Hip‐knee‐ankle angle (HKA), mechanical lateral distal femoral angle (mLDFA), medial proximal tibial angle (MPTA), joint line convergence angle (JLCA), medial posterior slope (mSlope), lateral posterior slope (lSlope), lateral proximal femoral angle (LPFA), tibial torsion (TT), femoral torsion (FT) and leg length (LL). Additionally, the Insall‐Salvati index (ISI) and the tibial tuberosity‐trochlear groove distance (TTTG) were measured as patellofemoral parameters [10]. The intra‐ and interobserver variation were expressed as the mean of the automatically calculated values.

### Statistics

Data collection and analysis were performed using Microsoft Excel (Redmond, Washington, USA, Version 16.79.1) and SPSS (IBM SPSS Statistics, Version 26). Descriptive statistics, including mean, SD and range, were calculated for bony landmarks, angles, and axes. Next to this, intra‐ and interobserver variability was measured by applying the formula mentioned above. Furthermore, the root mean square error (RMSE) was used as the quantitative value of the deviation between the observed and the mean value for angles and axes across both image modalities and observers, with low RMSE values indicating high agreement between both methods. The overall precision of CBCT and MSCT was tested for normal distribution using the Kolmogorov‐Smirnov test. Subsequently, a parametric t‐test for independent samples was performed to compare the two groups. A *p*‐value of 0.05 was considered statistically significant.

## RESULTS

**Figure 4:**
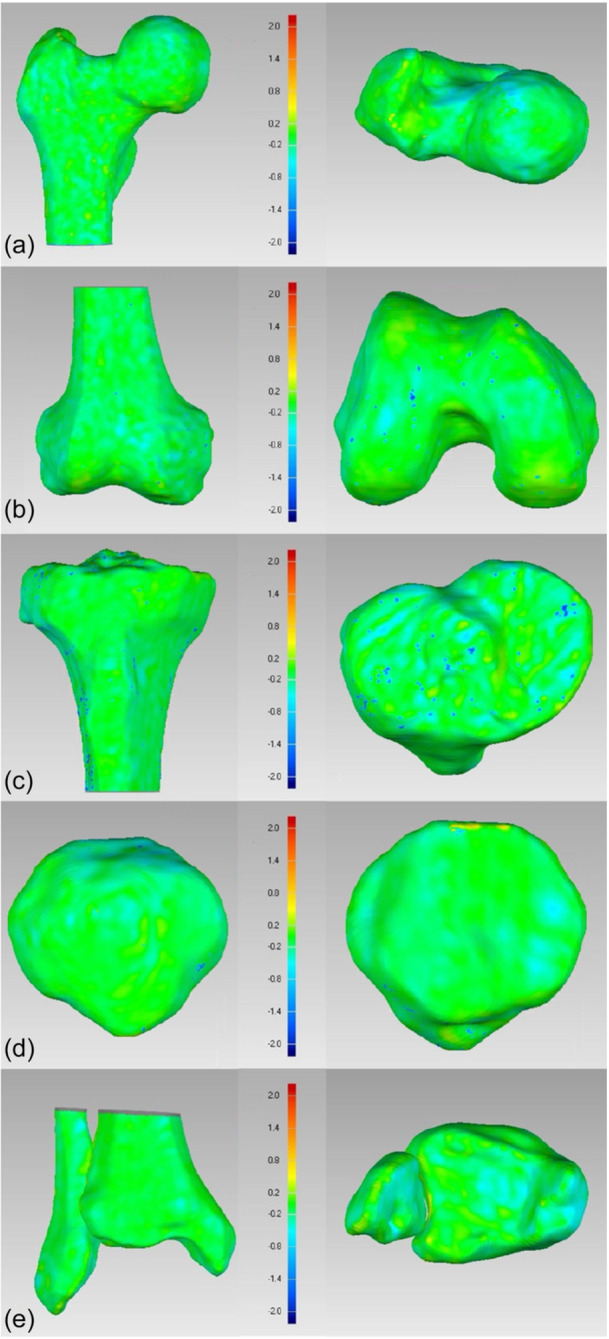
Surface comparison between 3D reconstruction of MSCT and CBCT of the (a) proximal femur (anterior and cranial view), (b) distal femur (anterior and caudal view), (c) proximal tibia (anterior and cranial view), (d) patella (anterior and posterior view), and (e) distal tibia and fibula (anterior and caudal view). The colouring describes the degree of deviation. The scale is presented in mm. CBCT, cone bean computed tomography; MSCT, multislice computed tomography.

The 3D reconstructions of both imaging methods were also checked for surface consistency. The surface comparison showed excellent agreement with most predominant deviation between 0.2 and −0.2 mm. Larger deviations above ±0.8 mm were limited to individual points and did not extend over larger areas (Figure 4).

The intra‐ and interobserver variability for each bony landmark in terms of the two imaging modalities is shown in Table 1. While the intraobserver deviation for all landmarks ranged from 0.39 mm (tibial lateral intercondylar tubercle) to 3.41 mm (tibial medial malleolus) in MSCT, the range in CBCT was 0.56 mm (tibial lateral intercondylar tubercle) to 2.39 mm (femoral medial condyle distal). The interobserver deviation exhibited a wider range, extending from 0.50 mm (tibial lateral intercondylar tubercle) to 4.43 mm (patella ridge high point) in MSCT and from 0.80 mm (patella distal pole) to 4.14 mm (tibial lateral condyle posterior) in CBCT.

**Table 1:** Intra‐ and interobserver variability of all landmarks in MSCT and CBCT.

	Interobserver deviation (mm)		Intraobserver deviation (mm)			
			1. Observer		2. Observer	
	MSCT	CBCT	MSCT	CBCT	MSCT	CBCT
	Mean (SD)	Mean (SD)	Mean (SD)	Mean (SD)	Mean (SD)	Mean (SD)
Femur						
Tip of greater trochanter (TGT)	**1.96** (0.85)	**1.68** (0.85)	**1.98** (0.23)	**1.24** (0.22)	**1.94** (0.61)	**2.14** (0.90)
Femoral head center (FHC)	**1.69** (0.50)	**1.22** (0.41)	**1.52** (0.29)	**1.23** (0.46)	**1.82** (0.57)	**1.19** (0.38)
Femoral neck center (FNC)	**1.86** (0.67)	**1.51** (0.71)	**1.50** (0.72)	**1.16** (0.35)	**1.66** (0.73)	**1.69** (0.85)
Femoral lateral condyle distal (FLCD)	**1.01** (0.46)	**1.37** (0.64)	**0.67** (0.34)	**0.94** (0.27)	**1.31** (0.37)	**1.80** (0.65)
Femoral medial condyle distal (FMCD)	**1.09** (0.43)	**1.72** (1.22)	**0.48** (0.11)	**0.89** (0.29)	**1.08** (0.40)	**2.39** (1.41)
Femoral lateral condyle posterior (FLCP)	**1.91** (1.43)	**1.55** (0.83)	**1.22** (0.43)	**1.03** (0.39)	**2.31** (1.92)	**1.54** (0.69)
Femoral medial condyle posterior (FMCP)	**1.52** (0.93)	**1.49** (0.47)	**1.10** (0.64)	**1.15** (0.38)	**1.79** (1.13)	**1.35** (0.20)
Femoral notch point (FNP)	**1.35** (0.95)	**1.50** (1.12)	**0.75** (0.42)	**0.78** (0.08)	**1.85** (1.04)	**1.95** (1.24)
Trochlear ridge medial point (TRMP)	**1.25** (0.62)	**0.98** (0.39)	**1.56** (0.51)	**0.85** (0.31)	**0.87** (0.38)	**0.86** (0.48)
Trochlear ridge lateral point (TRLP)	**2.44** (0.94)	**1.80** (1.26)	**1.98** (1.67)	**2.07** (1.58)	**1.08** (0.62)	**1.44** (0.70)
Trochlear groove central point (TGCP)	**1.62** (0.90)	**1.50** (0.74)	**1.77** (1.20)	**1.91** (0.53)	**0.52** (0.33)	**0.93** (0.41)
Tibia						
Tibial lateral condyle lateral (TLCL)	**1.35** (0.98)	**1.30** (0.90)	**0.43** (0.31)	**0.85** (0.22)	**1.93** (1.13)	**1.59** (0.99)
Tibial medial condyle medial (TMCM)	**1.42** (0.73)	**1.79** (0.99)	**1.23** (0.62)	**0.95** (0.45)	**1.53** (0.68)	**1.41** (0.68)
Tibial medial intercondylar tubercle (TMIT)	**1.00** (0.67)	**1.32** (0.37)	**0.67** (0.14)	**1.21** (0.43)	**1.12** (0.73)	**1.03** (0.57)
Tibial lateral intercondylar tubercle (TLIT)	**0.50** (0.23)	**0.88** (0.26)	**0.39** (0.13)	**0.56** (0.23)	**0.52** (0.27)	**0.95** (0.33)
Tibial knee center (TKC)	**0.67** (0.27)	**0.91** (0.46)	**0.48** (0.25)	**0.66** (0.10)	**0.56** (0.38)	**1.03** (0.59)
Tibial lateral condyle anterior (TLCA)	**1.41** (0.80)	**1.45** (0.67)	**0.84** (0.27)	**0.78** (0.22)	**1.36** (0.53)	**1.34** (0.29)
Tibial lateral condyle posterior (TLCP)	**3.47** (0.83)	**4.14** (1.66)	**0.86** (0.28)	**1.00** (0.26)	**1.04** (0.67)	**2.03** (1.29)
Tibial medial condyle anterior (TMCA)	**2.44** (1.94)	**1.86** (0.89)	**1.12** (0.86)	**0.77** (0.22)	**2.22** (1.92)	**2.29** (0.91)
Tibial medial condyle posterior (TMCP)	**2.14** (1.14)	**2.29** (0.60)	**1.02** (0.69)	**0.89** (0.44)	**2.23** (1.27)	**0.82** (0.26)
Tibial medial malleolus (TMM)	**3.03** (2.34)	**1.70** (0.96)	**1.69** (0.20)	**0.78** (0.37)	**3.41** (2.48)	**2.12** (0.91)
Tibial ankle center (TAC)	**1.88** (1.02)	**1.41** (0.57)	**1.61** (0.53)	**0.94** (0.65)	**2.10** (1.21)	**1.53** (0.49)
Tibial tuberosity point (TTP)	**1.97** (0.84)	**1.12** (0.48)	**1.20** (0.63)	**0.74** (0.23)	**1.89** (1.49)	**0.80** (0.48)
Fibula						
Fibular lateral malleolus (FLM)	**2.56** (1.10)	**1.80** (1.42)	**2.19** (1.16)	**0.98** (0.52)	**2.07** (0.80)	**2.30** (1.78)
Patella						
Patella proximal pole (PPP)	**1.33** (0.53)	**2.12** (0.65)	**1.15** (0.45)	**0.71** (0.19)	**1.39** (0.66)	**1.59** (1.02)
Patella distal pole (PDP)	**0.89** (0.34)	**0.80** (0.27)	**0.70** (0.29)	**0.78** (0.24)	**1.04** (0.34)	**0.81** (0.32)
Patella medial pole (PMP)	**1.18** (0.50)	**0.89** (0.40)	**0.80** (0.34)	**0.91** (0.26)	**1.06** (0.50)	**0.82** (0.42)
Patella lateral pole (PLP)	**0.90** (0.29)	**0.95** (0.41)	**0.71** (0.32)	**0.90** (0.41)	**0.92** (0.37)	**0.71** (0.29)
Patella ridge proximal pole (PRPP)	**1.16** (0.61)	**1.93** (1.61)	**0.88** (0.36)	**2.32** (1.76)	**1.43** (0.72)	**1.51** (0.78)
Patella ridge distal pole (PRDP)	**3.20** (0.85)	**2.72** (0.99)	**0.57** (0.20)	**0.95** (0.62)	**1.76** (1.07)	**1.21** (0.61)
Patella ridge high point (PRHP)	**4.43** (0.53)	**3.94** (1.08)	**0.74** (0.14)	**1.19** (0.31)	**1.34** (0.64)	**1.51** (0.56)

Abbreviations: CBCT, cone bean computed tomography; MSCT, multislice computed tomography; SD, standard deviation.

The precision of all landmarks averaged 1.38 mm (SD 0.63) in CBCT and 1.46 (SD 0.74) in MSCT with no statistically significant difference between the two groups (*p* = 0.444). Overall, landmark precision was less than 5 mm and on average 1.42 mm (SD 0.68 mm) for both imaging modalities.

**Figure 5:**
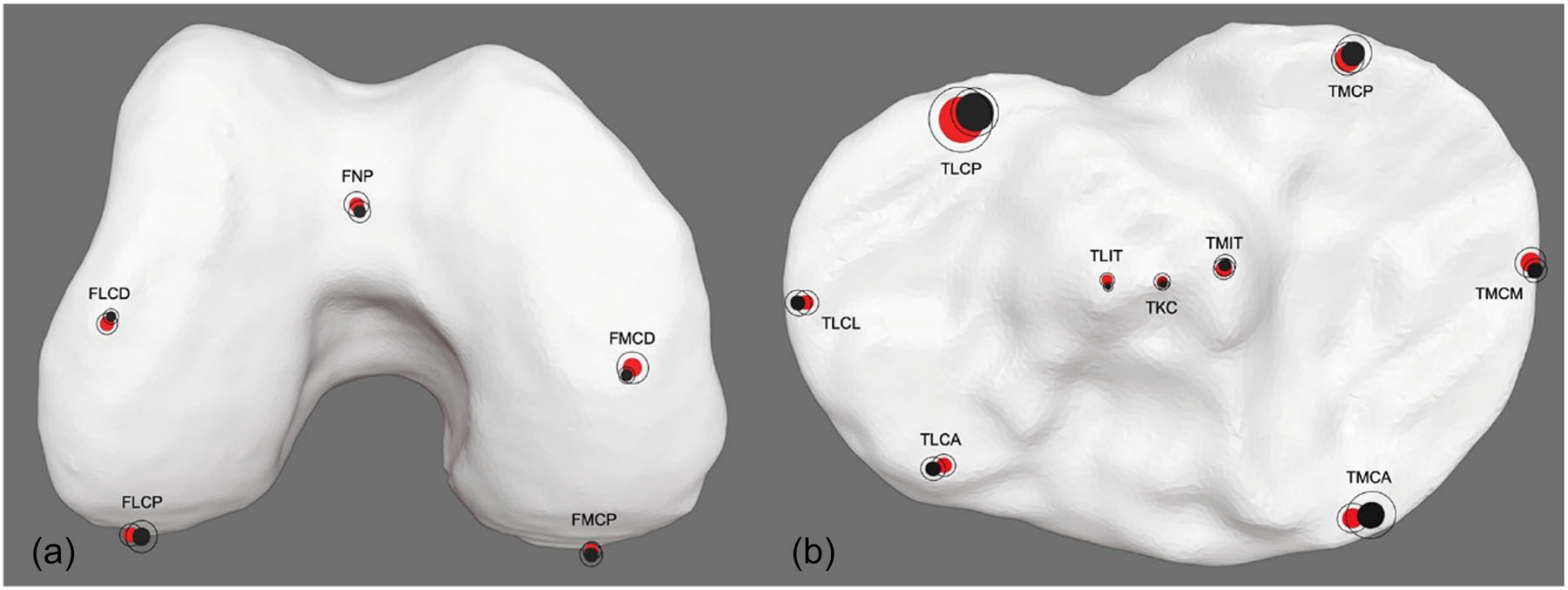
Bony landmarks of the distal femur (caudal to cranial view, a) and proximal tibia (cranial to caudal view, b) in a 3D reconstruction by CBCT. The filled areas represent the mean error (black for MSCT and red for CBCT), while the thin black circles indicate the mean error plus one standard deviation. CBCT, cone bean computed tomography; FLCD, femoral lateral condyle distal; FLCP, femoral lateral condyle posterior; FMCD, femoral medial condyle distal; FMCP, femoral medial condyle posterior; FNP, femoral notch point; MSCT, multislice computed tomography; TKC, tibial knee center; TLCA, tibial lateral condyle anterior; TLCL, tibial lateral condyle lateral; TLCP, tibial lateral condyle posterior; TLIT, tibial lateral intercondylar tubercle; TMIT, tibial medial intercondylar tubercle; TMCA, tibial medial condyle anterior; TMCM, tibial medial condyle medial; TMCP, tibial medial condyle posterior.

Upon closer examination of the landmarks on the distal femur and proximal tibia, the posterior lateral tibial condyle exhibited the greatest variability. However, when comparing the localization of detected landmarks between MSCT and CBCT, the circles representing the mean error for both imaging modalities consistently intersect, indicating that landmarks were identified in similar positions. This suggests a high level of agreement and comparable reliability for 3D landmark identification across both imaging modalities (Figure 5).

For angles and axes, the interobserver mean values in MSCT and CBCT differed by less than 1° in nearly all measurements. However, in the case of femoral torsion, the difference exceeded 2° (Table 2).

**Table 2:** Mean values of all angles, axes, patellofemoral values, and leg length measured in MSCT and CBCT.

			1. Observer		2. Observer	
	MSCT	CBCT	MSCT	CBCT	MSCT	CBCT
	Mean (SD)	Mean (SD)	Mean (SD)	Mean (SD)	Mean (SD)	Mean (SD)
Angles and axis (°)						
HKA	**176.49** (0.21)	**176.39** (0.19)	**176.38** (0.18)	**176.23** (0.11)	**176.60** (0.19)	**176.54** (0.10)
mLDFA	**88.78** (0.22)	**88.86** (0.17)	**88.90** (0.10)	**88.92** (0.13)	**88.66** (0.24)	**88.80** (0.20)
MPTA	**86.72** (0.56)	**86.02** (0.54)	**86.70** (0.35)	**85.98** (0.30)	**86.74** (0.75)	**86.07** (0.76)
JLCA	**1.45** (0.54)	**0.81** (0.54)	**1.43** (0.34)	**0.83** (0.27)	**1.48** (0.73)	**0.80** (0.77)
mSlope	**7.16** (0.72)	**7.62** (1.11)	**6.71** (0.56)	**6.62** (0.30)	**7.61** (0.58)	**8.61** (0.47)
lSlope	**9.32** (0.88)	**9.67** (0.87)	**8.61** (0.31)	**8.87** (0.24)	**10.03** (0.61)	**10.47** (0.23)
LPFA	**107.65** (0.67)	**107.75** (0.40)	**107.38** (0.55)	**107.68** (0.25)	**107.91** (0.73)	**107.82** (0.54)
Tibial torsion	**42.31** (3.08)	**42.89** (2.71)	**44.30** (2.06)	**44.41** (0.56)	**40.31** (2.66)	**41.37** (3.23)
Femoral torsion	**11.26** (1.68)	**9.10** (2.96)	**11.08** (2.19)	**10.25** (1.39)	**11.43** (1.22)	**7.95** (3.80)
Patellofemoral						
ISI	**0.96** (0.08)	**1.03** (0.18)	**0.92** (0.03)	**0.94** (0.02)	**1.05** (0.06)	**1.12** (0.22)
TTTG (mm)	**3.03** (1.05)	**3.56** (0.99)	**2.76** (0.89)	**3.95** (1.30)	**3.31** (1.22)	**3.18** (0.40)
Leg length (cm)	**76.76** (0.04)	**76.73** (0.05)	**76.77** (0.03)	**76.75** (0.03)	**76.74** (0.04)	**76.70** (0.06)

Abbreviations: CBCT, cone bean computed tomography; HKA, hip‐knee‐ankle angle; ISI, Insall–Salvati index; JLCA joint line convergence angle; LPFA, lateral proximal femoral anlge; mLDFA, mechanical lateral distal femoral angle; MPTA, medial proximal tibial angle; MSCT, multislice computed tomography; SD, standard deviation; TTTG, tibial tuberosity‐trochlear groove distance.

The RMSE for angles and axes in a combined group of CBCT and MSCT were less than 1° in most measurements. Exceptions were noted for tibial and femoral torsion, where the RMSE exceeded 2° (Table 3).

**Table 3:** Root mean square error (RMSE) for angles, axes and patellofemoral values in a combined group of MSCT and CBCT.

	Overall	1. Observer	2. Observer
	RMSE	RMSE	RMSE
Angles and axis (°)			
HKA	0.20	0.15	0.14
mLDFA	0.19	0.11	0.21
MPTA	0.63	0.47	0.75
JLCA	0.60	0.40	0.75
mSlope	0.91	0.40	0.69
lSlope	0.85	0.28	0.47
LPFA	0.53	0.41	0.58
Tibial torsion	2.77	1.35	2.70
Femoral torsion	2.52	1.69	3.06
Patellofemoral			
ISI	0.13	0.03	0.15
TTTG (mm)	1.00	1.16	0.81
Leg length (cm)	0.04	0.03	0.05

Abbreviations: CBCT, cone bean computed tomography; HKA, hip‐knee‐ankle angle; ISI, Insall–Salvati index; JLCA, joint line convergence angle; LPFA, lateral proximal femoral anlge; mLDFA, mechanical lateral distal femoral angle; MPTA, medial proximal tibial angle; MSCT, multislice computed tomography; TTTG, tibial tuberosity‐trochlear groove distance.

## DISCUSSION

The key finding of this study is the excellent level of agreement between 3D bony anatomy images of the lower extremity obtained from upright CBCT and MSCT. At the knee joint level, alignment parameters, and joint angles showed RMSE values of less than 1°, except for tibial and femoral torsion. However, torsional measurements have also shown higher variance in other studies, which supports the acceptability of our values [11, 28]. The differences in manual measurements of bony landmarks between the two imaging modalities were minor, consistently less than 5 mm, and within the range of variation seen with repeated manual measurements on the same 3D model [10, 11]. The slight discrepancies observed can be attributed primarily to the inherent variance associated with manual measurements on 3D anatomical models. Technological advancements, such as AI‐driven segmentation algorithms and landmark detection methods have the potential to reduce the discrepancies observed in manual segmentation [2, 13, 26]. Future integration of these innovations into both CBCT and MSCT could further improve accuracy and reduce operator‐dependent variability, making these modalities even more comparable for lower extremity assessments.

Consistency of surface reconstruction between the modalities was excellent, with most deviations being within ±0.2 mm. Larger deviations were limited to singular points, likely reflecting differences in the segmentation process. The accuracy of CBCT in capturing bony structures, particularly in comparison to MSCT, has been well‐established in other regions, especially the maxillofacial area [20]. Liang et al. reported deviations between 0.1–0.2 mm when comparing CBCT and MSCT‐based mandibular models and concluded that this represented an acceptable level of agreement [20]. Accordingly, our findings demonstrate a similarly high degree of concordance. However, the lower extremities present a unique challenge due to the larger region of interest.

Kuiper et al. conducted repeated manual landmark detection on 3D bone models derived from MSCT images of the lower limb, utilizing the landmarks defined by Fürmetz et al. [11, 18]. The authors described a mean difference for landmark positions of 2.01 mm (SD 1.64). In comparison, the present study demonstrated a mean difference of 1.46 mm (SD 0.74) and 1.38 (SD 0.63) using the same landmarks on 3D bone models generated from MSCT and CBCT scans, respectively. Moreover, the mean precision of all landmarks of both imaging modalities showed no significant difference (*p* = 0.444) in this study, highlighting their comparability.

Imaging protocols for the lower limb often focus on specific areas—hips, knees and ankles—to reduce radiation exposure. Recent advances in MSCT have introduced ultra‐low dose protocols, with median total dose equivalents below 0.2 mSv, comparable to a single pelvic radiograph [17]. These protocols provide images that are sufficient for assessing femoral and tibial torsion. However, the measurements were performed on axial slices and not on 3D models. Although 3D measurements based on low‐dose methods appear feasible, their use is not yet common due to a lack of studies confirming their applicability.

Furthermore, weight‐bearing effects, which play a critical role in assessing joint mechanics, cannot be accurately captured with supine MSCT. In contrast to standing posture, the ground reaction force acting on the knee joint, which is defined by Newton's third law as the opposing force exerted by the ground in response to body weight, is absent when the individual is in a supine position [5]. Previous CBCT studies have highlighted significant differences in knee joint alignment under weight‐bearing conditions, including parameters such as femorotibial rotation, TTTG, and medial femorotibial joint space width [12].

To date, no imaging modality provides comprehensive 3D visualization of the lower limb alignment under full weight‐bearing conditions including the patellofemoral anatomy. The results of this phantom study, however, suggest that upright CBCT offers a high degree of accuracy when compared with MSCT for lower extremity imaging.

## LIMITATIONS

This study uses a bone model phantom, which, while valuable for establishing a baseline comparison between CBCT and MSCT, does not fully replicate the complexity of in vivo imaging. The study is further limited by using a model from only one CT data set, potential inaccuracies in manual measurements affecting comparability, and the inability to assess radiation exposure. Next to this, the observers could not be blinded to the type of scan while landmark detection, as the differences between the imaging modalities were readily apparent. The study design did not account for weight‐bearing effects due to the use of a fixed model. Studies exploring the impact of soft tissue on imaging accuracy and radiation dose are needed to provide a more comprehensive understanding of these modalities' applicability to clinical practice.

## CONCLUSION

This laboratory study demonstrates excellent agreement between CBCT and MSCT for 3D imaging of the lower limb in the assessment of alignment and joint level metrics. By offering the same accuracy as MSCT while allowing for weight‐bearing during image acquisition, CBCT may have the potential to enhance preoperative planning, simulation, and surgical outcome through precise 3D assessment of lower limb alignment. Further studies are needed to confirm these results in the presence of surrounding soft tissue.

## AUTHOR CONTRIBUTIONS

Julian Fürmetz, Julius Watrinet, and Philipp Blum planned the study. Marianne Hollensteiner and Philipp Blum built the limb phantom. Michael Scherr, Michael Seidenbusch, and Marcus Treitl helped carrying out the scans. Julius Watrinet and Philipp Blum performed leg axis analysis. Julian Fürmetz, Peter Augat, and Philipp Blum contributed to the interpretation of the results. Julian Fürmetz and Philipp Blum took the lead in writing the manuscript. Julius Watrinet, Marianne Hollensteiner, Peter Augat, and Fabian Stuby helped to shape the final manuscript. All authors provided critical feedback and agreed to the published version of this manuscript.

## CONFLICT OF INTEREST STATEMENT

The authors declare no conflicts of interest.

## ETHICS STATEMENT

Institutional Review Board approval was not required because no human participants were involved in this descriptive laboratory study. Written informed consent was not required for this study because the used CT scan has been deidentified.

## DECLARATION

Multitom Rax with the 3D option is not available in all countries. Its future availability cannot be guaranteed.

## Data Availability

The data presented in this study are available on request from the corresponding author.

## References

[1] Ahrend M‐D , Baumgartner H , Ihle C , Histing T , Schröter S , Finger F . Influence of axial limb rotation on radiographic lower limb alignment: a systematic review. Arch Orthop Trauma Surg. 2022;142(11):3349–3366. 34596760 10.1007/s00402-021-04163-w PMC9522705

[2] Archer H , Reine S , Xia S , Vazquez LC , Ashikyan O , Pezeshk P , et al. Reliability assessment of leg length and angular alignment on manual reads versus artificial intelligence‐generated lower extremity radiographic measurements. Clin Imaging. 2024;113:110233. 10.1016/j.clinimag.2024.110233 39029361

[3] Buck FM , Guggenberger R , Koch PP , Pfirrmann CWA . Femoral and tibial torsion measurements with 3D models based on low‐dose biplanar radiographs in comparison with standard CT measurements. Am J Roentgenol. 2012;199(5):W607–W612. 23096205 10.2214/AJR.11.8295

[4] Chaibi Y , Cresson T , Aubert B , Hausselle J , Neyret P , Hauger O , et al. Fast 3D reconstruction of the lower limb using a parametric model and statistical inferences and clinical measurements calculation from biplanar X‐rays. Comput Methods Biomech Biomed Engin. 2012;15(5):457–466. 21229412 10.1080/10255842.2010.540758

[5] Colyn W , Vanbecelaere L , Bruckers L , Scheys L , Bellemans J . The effect of weight‐bearing positions on coronal lower limb alignment: a systematic review. Knee. 2023;43:51–61. 37271072 10.1016/j.knee.2023.05.004

[6] Degen N , Fürmetz J , Wolf F , Euler E , Thaller P . Medial proximal femoral angle better than neck‐shaft angle? Influence of rotation on the anteroposterior radiograph. J Hip Surg. 2017;1(3):146–151.

[7] Degen N , Sass J , Jalali J , Kovacs L , Euler E , Prall WC , et al. Three‐dimensional assessment of lower limb alignment: reference values and sex‐related differences. Knee. 2020;27(2):428–435. 31806504 10.1016/j.knee.2019.11.009

[8] Fedorov A , Beichel R , Kalpathy‐Cramer J , Finet J , Fillion‐Robin J‐C , Pujol S , et al. 3D Slicer as an image computing platform for the Quantitative Imaging Network. Magn Reson Imaging. 2012;30(9):1323–1341. 22770690 10.1016/j.mri.2012.05.001 PMC3466397

[9] Folinais D , Thelen P , Delin C , Radier C , Catonne Y , Lazennec JY . Measuring femoral and rotational alignment: EOS system versus computed tomography. Orthop Traumatol Surg Res. 2013;99(5):509–516. 23877073 10.1016/j.otsr.2012.12.023

[10] Fürmetz J , Daniel T , Sass J , Bergsträßer M , Degen N , Suero E , et al. Three‐dimensional assessment of patellofemoral anatomy: reliability and reference ranges. Knee. 2021;29:271–279. 33677151 10.1016/j.knee.2021.02.016

[11] Fürmetz J , Sass J , Ferreira T , Jalali J , Kovacs L , Mück F , et al. Three‐dimensional assessment of lower limb alignment: accuracy and reliability. Knee. 2019;26(1):185–193. 30473372 10.1016/j.knee.2018.10.011

[12] Hirschmann A , Buck FM , Fucentese SF , Pfirrmann CWA . Upright CT of the knee: the effect of weight‐bearing on joint alignment. Eur Radiol. 2015;25(11):3398–3404. 25929941 10.1007/s00330-015-3756-6

[13] Jo C , Hwang D , Ko S , Yang MH , Lee MC , Han H‐S , et al. Deep learning‐based landmark recognition and angle measurement of full‐leg plain radiographs can be adopted to assess lower extremity alignment. Knee Surg Sports Traumatol Arthrosc. 2023;31(4):1388–1397. 36006418 10.1007/s00167-022-07124-x

[14] Jud L , Roth T , Fürnstahl P , Vlachopoulos L , Sutter R , Fucentese SF . The impact of limb loading and the measurement modality (2D versus 3D) on the measurement of the limb loading dependent lower extremity parameters. BMC Musculoskelet Disord. 2020;21(1):418. 32605616 10.1186/s12891-020-03449-1 PMC7329436

[15] Kai S , Sato T , Koga Y , Omori G , Kobayashi K , Sakamoto M , et al. Automatic construction of an anatomical coordinate system for three‐dimensional bone models of the lower extremities‐‐pelvis, femur, and tibia. J Biomech. 2014;47(5):1229–1233. 24456665 10.1016/j.jbiomech.2013.12.013

[16] Kannan A , Hawdon G , McMahon S . Effect of flexion and rotation on measures of coronal alignment after TKA. J Knee Surg. 2012;25(5):407–410. 23150351 10.1055/s-0032-1313756

[17] Keller G , Götz S , Kraus MS , Grünwald L , Springer F , Afat S . Radiation dose reduction in CT torsion measurement of the lower limb: introduction of a new ultra‐low dose protocol. Diagnostics. 2021;11(7):1209. 34359292 10.3390/diagnostics11071209 PMC8304839

[18] Kuiper RJA , Seevinck PR , Viergever MA , Weinans H , Sakkers RJB . Automatic assessment of lower‐limb alignment from computed tomography. J Bone Jt Surg. 2023;105(9):700–712. 10.2106/JBJS.22.00890 36947661

[19] Lee HS , Hwang S , Kim S‐H , Joon NB , Kim H , Hong YS , et al. Automated analysis of knee joint alignment using detailed angular values in long leg radiographs based on deep learning. Sci Rep. 2024;14(1):7226. 38538685 10.1038/s41598-024-57887-1 PMC10973521

[20] Liang X , Lambrichts I , Sun Y , Denis K , Hassan B , Li L , et al. A comparative evaluation of Cone Beam Computed Tomography (CBCT) and Multi‐Slice CT (MSCT). Part II: on 3D model accuracy. Eur J Radiol. 2010;75(2):270–274. 19423257 10.1016/j.ejrad.2009.04.016

[21] Maderbacher G , Baier C , Benditz A , Wagner F , Greimel F , Grifka J , et al. Presence of rotational errors in long leg radiographs after total knee arthroplasty and impact on measured lower limb and component alignment. Int Orthop. 2017;41(8):1553–1560. 28144722 10.1007/s00264-017-3408-3

[22] Pflüger P , Hodel S , Zimmermann SM , Knechtle S , Vlachopoulos L , Fucentese SF . The coronal alignment differs between two‐dimensional weight‐bearing and three‐dimensional nonweight bearing planning in total knee arthroplasty. J Exp Orthop. 2024;11(1):e12007. 38455454 10.1002/jeo2.12007 PMC10885761

[23] Predescu V , Grosu A‐M , Gherman I , Prescura C , Hiohi V , Deleanu B . Early experience using patient‐specific instrumentation in opening wedge high tibial osteotomy. Int Orthop. 2021;45(6):1509–1515. 33580315 10.1007/s00264-021-04964-z

[24] Rosso F , Rossi R , Neyret P , Śmigielski R , Menetrey J , Bonasia DE , et al. A new three‐dimensional patient‐specific cutting guide for opening wedge high tibial osteotomy based on ct scan: preliminary in vitro results. J Exp Orthop. 2023;10(1):80. 37556100 10.1186/s40634-023-00647-3 PMC10412513

[25] Schock J , Truhn D , Abrar DB , Merhof D , Conrad S , Post M , et al. Automated analysis of alignment in long‐leg radiographs by using a fully automated support system based on artificial intelligence. Radiol Artif Intell. 2021;3(2):200198. 10.1148/ryai.2020200198 PMC8043357 33937861

[26] Van Den Borre I , Peiffer M , Huysentruyt R , Huyghe M , Vervelghe J , Pizurica A , et al. Development and validation of a fully automated tool to quantify 3D foot and ankle alignment using weight‐bearing CT. Gait Posture. 2024;113:67–74. 38850852 10.1016/j.gaitpost.2024.05.029

[27] Victor J , Van Doninck D , Labey L , Innocenti B , Parizel PM , Bellemans J . How precise can bony landmarks be determined on a CT scan of the knee? Knee. 2009;16(5):358–365. 19195896 10.1016/j.knee.2009.01.001

[28] Yan W , Xu X , Xu Q , Yan W , Sun Z , Jiang Q , et al. Femoral and tibial torsion measurements based on EOS imaging compared to 3D CT reconstruction measurements. Ann Transl Med. 2019;7(18):460. 31700896 10.21037/atm.2019.08.49 PMC6803189

[29] Zahn RK , Renner L , Perka C , Hommel H . Weight‐bearing radiography depends on limb loading. Knee Surg Sports Traumatol Arthrosc. 2019;27(5):1470–1476. 29992465 10.1007/s00167-018-5056-6

[30] Zhang J , Ndou WS , Ng N , Gaston P , Simpson PM , Macpherson GJ , et al. Robotic‐arm assisted total knee arthroplasty is associated with improved accuracy and patient reported outcomes: a systematic review and meta‐analysis. Knee Surg Sports Traumatol Arthrosc. 2022;30(8):2677–2695. 33547914 10.1007/s00167-021-06464-4 PMC9309123

